# Endoplasmic Reticulum (ER) Stress and Endocrine Disorders

**DOI:** 10.3390/ijms18020382

**Published:** 2017-02-11

**Authors:** Daisuke Ariyasu, Hiderou Yoshida, Yukihiro Hasegawa

**Affiliations:** 1 Division of Developmental Genetics, Institute of Resource Development and Analysis, Kumamoto University, Kumamoto 860-0811, Japan; daisukeariyasu@gmail.com; 2 Department of Biochemistry and Molecular Biology, Graduate School of Life Science, University of Hyogo, Hyogo 678-1297, Japan; 3 Division of Endocrinology and Metabolism, Tokyo Metropolitan Children’s Medical Center, Tokyo 183-8561, Japan

**Keywords:** endoplasmic reticulum stress, endocrine disorder, PKR-like endoplasmic reticulum kinase (PERK), activating transcription factor 6 (ATF6), inositol requirement 1 (IRE1), old astrocyte specifically induced substance (OASIS) family, chemical chaperone

## Abstract

The endoplasmic reticulum (ER) is the organelle where secretory and membrane proteins are synthesized and folded. Unfolded proteins that are retained within the ER can cause ER stress. Eukaryotic cells have a defense system called the “unfolded protein response” (UPR), which protects cells from ER stress. Cells undergo apoptosis when ER stress exceeds the capacity of the UPR, which has been revealed to cause human diseases. Although neurodegenerative diseases are well-known ER stress-related diseases, it has been discovered that endocrine diseases are also related to ER stress. In this review, we focus on ER stress-related human endocrine disorders. In addition to diabetes mellitus, which is well characterized, several relatively rare genetic disorders such as familial neurohypophyseal diabetes insipidus (FNDI), Wolfram syndrome, and isolated growth hormone deficiency type II (IGHD2) are discussed in this article.

## 1. Introduction

The endoplasmic reticulum (ER) is the organelle where secretory and membrane proteins are synthesized. Nascent proteins synthesized in the ER are unfolded and structurally corrected by ER chaperones. Only properly folded proteins are transported to the Golgi apparatus and are able to function as secretory or membrane proteins. Misfolded proteins are retained within the ER and degraded by ER-associated degradation (ERAD) [1].

ER chaperones and ERAD capacity can cope with a certain number of unfolded proteins constantly produced under non-stress conditions. However, circumstances such as an increased demand for proteins or the presence of genetic abnormalities increase the amount of unfolded proteins in cells, which overwhelms the capacities of the ER chaperones and ERAD, leading to cell toxicity and death. Eukaryotic cells have a defense system, the “unfolded protein response” (UPR) or “the ER stress response”, which protects cells from such situations [2].

Apoptotic events driven by ER stress have been shown to be associated with disease in humans. Generally, neurons are the most susceptible to ER stress-mediated apoptosis because they cannot reproduce. Studies have revealed that the ER stress response is involved in different neurodegenerative disorders such as Alzheimer’s disease [3], Parkinson’s disease [4], and polyglutamine diseases [5].

The expression of peptide hormones can cause ER stress to cells because they often have complex three-dimensional protein structures, including disulfide bonds, and their requisite availability changes rapidly, which requires cells to adapt to protein demand by expanding ER membranes in a short period [6,7]. Therefore, endocrine cells may be particularly susceptible to ER stress. For example, insulin-resistant peripheral tissues force pancreatic β-cells to increase proinsulin production, which leads to an increase in misfolded proinsulin in the ER, causing ER stress [6]. Several pathological conditions, such as insulin resistance and insulin gene mutations, increase ER stress in pancreatic β-cells, which leads to hyperglycemia [8]. Arginine-vasopressin (AVP) is a hormone produced in the hypothalamus, and is derived from the prohormone AVP-neurophysin II (AVP-NPII) by processing. AVP-NPII has seven disulfide bonds [9], and *NPII* gene mutations, which affect cysteine residues and alter protein structure, are the major cause of ER stress-mediated impaired secretion of AVP [10]. Recent studies have revealed the involvement of ER stress in other endocrine disorders, including growth hormone deficiency. In this review, we focus on ER stress-mediated endocrine disorders.

## 2. Unfolded Protein Responses in Mammals

First, we describe the unfolded protein response in mammals. The PKR-like endoplasmic reticulum kinase (PERK), activating transcription factor 6 (ATF6), and inositol requirement 1 (IRE1) pathways are well characterized as the three major UPR pathways in mammals. These work together for the coordinated repression of general translation and the activation of the expression of UPR chaperones and ERAD.

### 2.1. PERK Pathway

**Figure 1:**
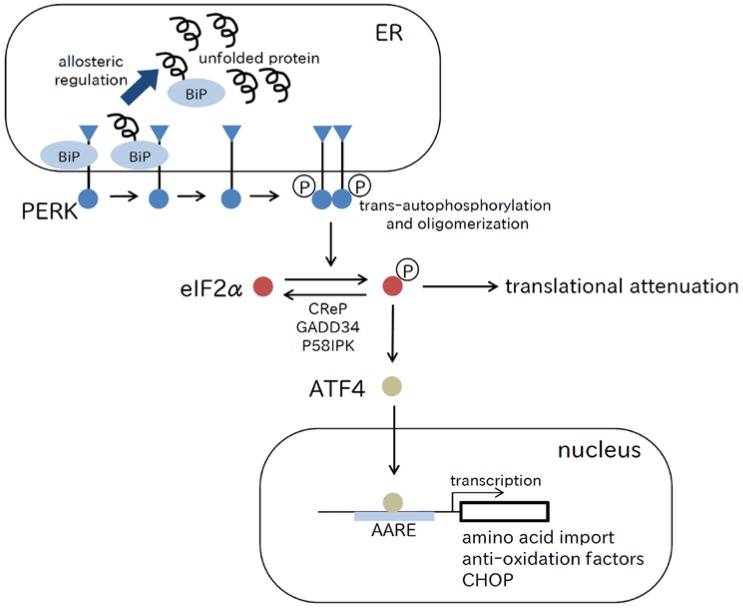
The PERK pathway. PERK oligomerizes and *trans*-autophosphorylates by detecting unfolded proteins in the ER, inactivates eIF2α by phosphorylation, and induces translation of ATF4. According to the current allosteric model of UPR induction, the ATPase domain of BiP interacts with the luminal domain of PERK that dissociates when an unfolded protein binds to the canonical substrate binding domain of BiP. ATF4 enhances transcription of genes involved in amino acid transport, oxidative stress, and apoptosis. Phosphorylated eIF2α is dephosphorylated by CReP, GADD34, and P58IPK. AARE, amino acid response element; ATF4, activating transcription factor 4; CHOP, C/EBP-homologous protein; CReP, constitutive repressor of eIF2α phosphorylation; eIF2α, eukaryotic translational initiation factor 2; GADD34, growth arrest and DNA damage 34; PERK, PKR-like endoplasmic reticulum kinase; P58IPK, 58 kDa-inhibitor of protein kinase.

PERK is localized on the ER membrane, and detects the accumulation of unfolded proteins in the ER lumen. In the presence of ER stress, PERK is activated by *trans*-autophosphorylation and oligomerization. Activated PERK inactivates eukaryotic translational initiation factor 2 (eIF2α), which is the α subunit of eukaryotic initiation factor 2 by phosphorylation, leading to general inhibition of protein translation [11] (Figure 1). Although it remains obscure how the luminal domain of PERK detects unfolded protein accumulation in the ER, an important allosteric model of UPR induction was recently proposed in which the ATPase domain of the ER chaperone, immunoglobulin heavy-chain binding protein (BiP), interacts with the luminal domains of PERK and IRE1 (described below), which dissociates when an unfolded protein binds to the canonical substrate binding domain of BiP [12]. Salubrinal [13], guanabenz [14], and sephin1 [15], which inhibit dephosphorylation of eIF2α, protect cells from apoptosis and suggest that inhibition of protein translation by PERK has a cytoprotective effect. In contrast, phosphorylated eIF2α activates translation of genes that have short upstream open reading frames in their 5′-untranslated region, which leads to increased translation of genes such as transcription factor activating transcription factor 4 (ATF4). ATF4 upregulates genes involved in amino acid import, metabolism, and resistance to oxidative stress, which also confer a cytoprotective effect in the early phase of ER stress [16,17]. However, ATF4 activates C/EBP-homologous protein (CHOP), which is a proapoptotic transcription factor (discussed below), in the later phase of ER stress. CHOP inhibits function of B cell leukemia 2 (BCL2) family members, which protect cells from apoptosis, and therefore leads to cell death.

Thus, the PERK pathway has an opposing effect on cellular conditions being both cytoprotective and apoptotic. Currently, PERK is believed to have a role in determining cell fate under ER stress [18]. Recently, it was demonstrated that the eIF2α-ATF4 pathway induced autophagy [19]. It is proposed that autophagy induced by the PERK pathway is a factor in the determination of cell fate by PERK [20].

### 2.2. ATF6 Pathway

**Figure 2:**
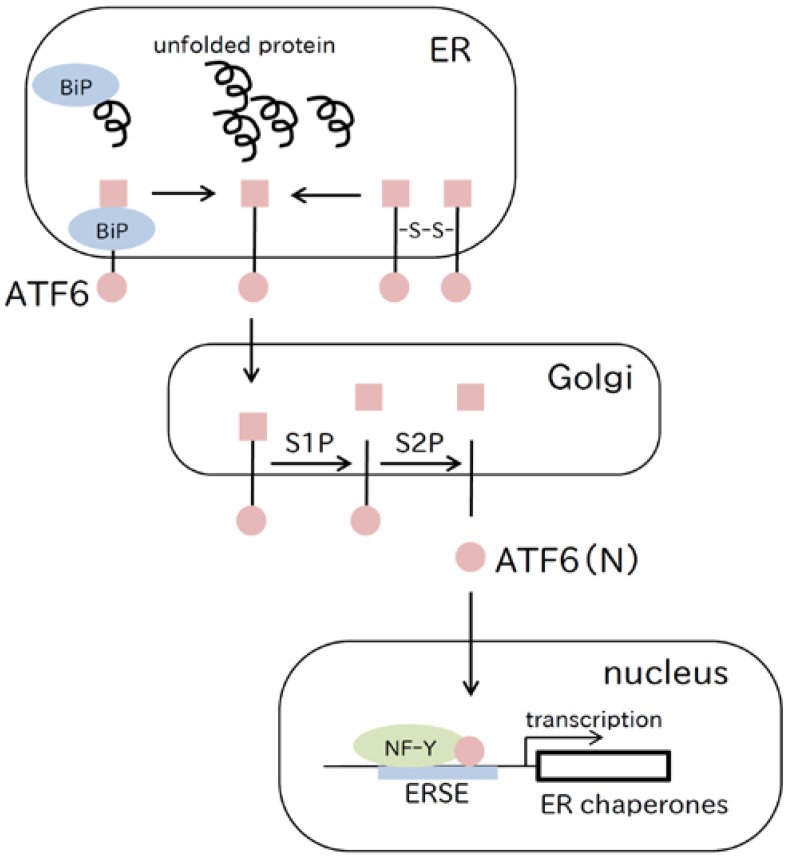
The ATF6 pathway. ATF6 detects unfolded proteins in the ER, and translocates to the Golgi apparatus. In the Golgi, ATF6 is cleaved by site 1 protease (S1P) and site 2 protease (S2P), and the N-terminal portion, ATF6(N), is translocated to the nucleus. ATF6(N) binds to the ERSE forming a heterodimer with NF-Y and enhances transcription of ER chaperone genes.

ATF6 is an ER membrane-located sensor protein, which also functions as a bZip transcription factor that enhances expression of ER chaperone genes. By sensing unfolded proteins in the ER, ATF6 translocates by coat protein complex II (COPII) vesicle transport from the ER to the Golgi, where it undergoes regulated intramembrane proteolysis (RIP) by Site-1 and Site-2 proteases [21,22]. Currently, there are two working models of the stress sensing mechanisms of ATF6. The first model is that BiP dissociation from ATF6 upon ER stress uncovers the Golgi localization signal of ATF6, which leads to ATF6 translocation to the Golgi [22,23,24]. The second model is that the luminal domains of ATF6 form dimers or oligomers by intermolecular disulfide bonds under normal conditions in the ER. In response to ER stress, the disulfide bonds of ATF6 are cleaved and the resultant monomeric ATF6 translocates to the Golgi [25,26,27] (Figure 2).

The N-terminal bZip domain of ATF6(N) released from the Golgi membrane by RIP enters the nucleus, and upregulates the expression of genes through the ER stress response element (ERSE) [28]. Many target genes of ATF6(N) are involved in ER quality control, including ER chaperone genes and folding enzyme genes [29,30,31,32]. There are two ubiquitously expressed isoforms of ATF6 in mammals, ATF6α and ATF6β [33,34]. ATF6α knockout (KO) mice and ATF6β KO mice are susceptible to ER stress, but are completely viable [35,36]. On the other hand, an ATF6α/β double KO causes embryonic lethality in mice, the reason of which is unknown. However, using medaka fish, it was demonstrated that embryonic lethality in ATF6α/β double KO medaka was caused by impaired notochord development [37]. Medaka fish may be a useful vertebrate model organism because the medaka genome encodes five UPR transducers and mammalian UPR signaling pathways are well conserved [38].

Recently, ceapins, which are a class of pyrazole amides, have been shown to be selective inhibitors of ATF6α signaling that do not inhibit Golgi proteases or other UPR branches [39,40].

### 2.3. IRE1 Pathway

**Figure 3:**
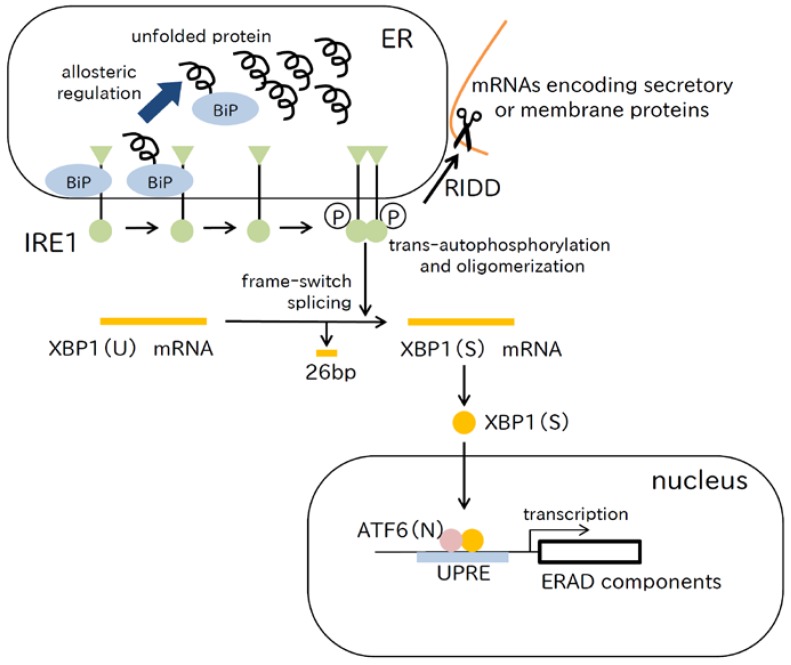
The IRE1 pathway. Using the allosteric model of UPR induction, the mechanism of detecting unfolded proteins by the luminal domain of IRE1 is shown. The ATPase domain of BiP interacts with the luminal domain of IRE1, which dissociates when an unfolded protein binds to the canonical substrate binding domain of BiP. IRE1 is activated by oligomerization and *trans*-autophosphorylation. Next, IRE1 converts XBP1(U) mRNA into XBP1(S) mRNA by frame-switch splicing, which leads to XBP1(S) production. XBP1(S) translocates to the nucleus, and binds to the UPRE forming a heterodimer with ATF6(N), which results in enhancement of gene expression encoding ERAD components. IRE1 RNase activity degrades mRNAs associated with the ER membrane, thereby unburdening the protein load through regulated IRE1-dependent decay of mRNA (RIDD).

IRE1 is the third sensing protein in the ER membrane, which has orthologs in all eukaryotes [41]. The luminal domain of IRE1 is similar to that of PERK and is involved in the detection of unfolded proteins, whereas the cytoplasmic domain contains a kinase domain and an ribonuclease (RNase) domain [2] (Figure 3). In the presence of ER stress, IRE1 is activated by *trans*-autophosphorylation and oligomerization [42]. In yeast Ire1, recent evidence indicates that downstream activation of Ire1 is influenced not only by BiP dissociation, but also by binding to unfolded proteins [43,44]. Direct binding of the Ire1 luminal domain to unfolded protein accumulated in the ER is not in line with the allosteric model of UPR induction previously described [12], and further study is needed.

Activated IRE1 converts XBP1 pre-mRNA (XBP1(U) mRNA) into mature XBP1 mRNA (XBP1(S) mRNA) by an unconventional splicing reaction. XBP1(U) mRNA has the DNA binding domain and the transcriptional activation domain in different reading frames. The unconventional splicing of XBP1(U) mRNA removes a 26-nt intron that results in translation of active XBP1(S), which has both domains in the same reading frame. XBP1(S) protein translocates to the nucleus, and binds to the unfolded protein response element (UPRE) to enhance expression of genes involved in ERAD by forming a heterodimer with ATF6(N) [45]. IRE1 recognizes the specific stem-loop RNA structure of XBP1(U) mRNA for splicing. XBP1(U) mRNA forms a complex with the nascent XBP1(U) polypeptide chain and a ribosome, which tethers and stabilizes XBP1(U) mRNA on the ER membrane, and thereby, leads to efficient splicing by IRE1 [46]. Translational pausing of XBP1(U) mRNA is mediated by the evolutionarily conserved peptide module at the carboxyl terminus, which is required for efficient targeting to the ER membrane and splicing of XBP1(U) mRNA by IRE1 [47]. In addition, XBP1 is regulated at the protein level. For example, XBP1(U) protein forms a complex with XBP1(S) protein, and undergoes rapid proteasome degradation under non-stress conditions, which shuts off the transcription of target genes of XBP1(S) during the recovery phase of ER stress [48]. It was also reported that the small ubiquitin-like modifier (SUMO) conjugase ubiquitin-conjugating enzyme 9 (UBC9) specifically binds to XBP1(S) and increases its stability [49].

IRE1 RNase activity is involved in a mechanism termed RIDD (regulated IRE1-dependent decay of mRNA), which selectively degrades ER-associated mRNAs coding secretory or membrane proteins, the purpose of which is to unburden the protein load of the ER [50,51]. mRNAs coding ER chaperones that maintain ER function avoid RIDD by an unknown mechanism, and it remains unclear how IRE1 recognizes RIDD targets. The consensus sequence of IRE1 mRNA cleavage sites is CUGCAG in the RNA stem-loop structure [52]; however, some mammalian RIDD target genes do not have this consensus sequence. XBP1 mRNA splicing is transiently activated specifically during the adaptive phase of ER stress. In contrast, RIDD is believed to be a constitutively active mechanism that develops with ER stress intensity and/or duration, which ultimately leads to cell death [53,54,55].

Recently, a novel characteristic of IRE1, which prevents ER membrane permeabilization and cell death, was reported. The leakage of ER luminal proteins is not because of ERAD, and ER membrane proteins escape this mechanism. This mechanism is exerted through the IRE1 kinase domain and the Jun N-terminal kinase (JNK) pathway (described below) [56].

### 2.4. Phase Shift of the ER Stress Pathways

The three major pathways described are temporally regulated. Upon ER stress, inhibition of protein translation by PERK is the first triggered among the three because phosphorylation of eIF2α is the sole necessary step. However, this step is transient and cells have to adapt to sustained ER stress. Therefore, induction of ER chaperones by ATF6 occurs next because it takes time to translocate ATF6 to the Golgi to be cleaved by RIP, to translocate the cleaved ATF6(N) to the nucleus, and to produce ER chaperones. ER chaperones are effectively translated after dephosphorylation of eIF2α and inactivation of the PERK pathway. The IRE1-XBP1 pathway is activated last because it requires cells to splice XBP1(U) mRNA, produce XBP1(S) protein, and translocate XBP1(S) to the nucleus to enhance expression of genes involved in ERAD. However, once the IRE1-XBP1 pathway is activated, XBP1(S) can activate its own expression by binding to ERSE in the promoter region of the *XBP1* gene, which is functionally sustainable as long as unfolded proteins are present in the ER [57]. The IRE1-XBP1 pathway acts more cytoprotectively since ERAD induced by the IRE1-XBP1 pathway can degrade unfolded proteins that cannot be refolded by ER chaperones, which are induced by the ATF6 pathway.

Furthermore, these three pathways are dependent on each other. For example, ATF6(N) binds to ERSE, which is located within the promoter of the *CHOP* gene that is downstream of PERK, and the *XBP1* gene, which is downstream of IRE1 [45,58]. Moreover, ATF4, which is activated downstream of PERK, was reported to activate the IRE1-XBP1 signal [59].

### 2.5. Apoptosis-Inducing Pathways

Mammalian cells induce apoptotic pathways when ER stress is not alleviated by the unfolded protein response (previously described). Three well-characterized cascades that cause apoptosis are described below.

CHOP is a transcription factor that is induced by ER stress. ATF4 and ATF6(N) bind to the amino acid response element (AARE) and to the ERSE in the promoter of the mammalian CHOP gene respectively, to enhance its expression [58,60,61]. CHOP induces expression of proapoptotic factors such as death receptor 5 (DR5), growth arrest and DNA damage 34 (GADD34), and ER oxidoreductin (ERO1) [62,63].

Activated IRE1 forms a complex with tumor necrosis factor receptor-associated factor2 (TRAF2) and apoptosis signal-regulating kinase 1 (ASK1), which phosphorylates JNK and leads to apoptosis [64,65]. IRE1 KO cells or ASK1 KO cells are resistant to JNK activation and apoptosis by ER stress, whereas TRAF2 KO cells are more susceptive to ER stress [66], which is not consistent with the notion that the IRE1–TRAF2–ASK1 complex induces apoptosis.

Caspases are well-characterized as components of the ER stress-specific apoptotic cascade. Caspase-12 in rodents and caspase-4 in humans activate the caspase-3 and caspase-9 mediated apoptotic pathways [67,68,69]. Casp12 KO mice are resistant to ER stress-mediated apoptosis, but are sensitive to other apoptotic signals [70,71].

### 2.6. ER Stress-Independent Functions of the UPR

Recent evidence shows that the UPR can also be activated by plasma membrane signaling in the absence of ER stress [72]. Toll-like receptors (TLR) are well-characterized pathogen-recognition receptors. TLR2 and TLR4 specifically activate IRE1α and XBP1 in the absence of ER stress, which leads to optimal and sustained production of proinflammatory cytokines in macrophages [73,74]. It has also been shown that well-known XBP1 maturation-mediated plasma cell differentiation is initiated by B-cell receptor signaling in a stress-independent manner [75,76]. In pancreatic β-cells, high glucose has been shown to be a physiological activator of IRE1α, suggesting that IRE1α monitors glucose fluctuations to regulate proinsulin production in the absence of ER stress [77]. In endothelial cells, vascular endothelial growth factor (VEGF) signals activate UPR mediators without accumulation of unfolded proteins in the ER [78]. These findings indicate that UPR signaling modules may have ER stress-independent functions.

### 2.7. OASIS Family

Recently, the old astrocyte specifically induced substance (OASIS) family of bZip transcription factors has been reported, which is an additional family of ER stress transducers. The five OASIS family members identified to date are Luman/CREB3 [79], OASIS/CREB3L1 [80], BBF2H7/CREB3L2 [81], CREBH/CREB3L3 [82], and CREB4/AIbZIP/CREB3L4/TISP40 [83,84]. They all share characteristics that are very similar to those of ATF6, such as having a transmembrane domain, a bZip domain, and a transcriptional activation domain, and that they undergo RIP in the Golgi apparatus [85]. Active N-terminal fragments of all OASIS members translocate to the nucleus and activate transcription of target genes. They are distinguishable by the conserved 30 amino acid region adjacent to the bZip domain (Adjacent to BZip or ATB domain), which other bZip transcription factors do not have, and the function of which is still to be elucidated [86]. OASIS family members are unique in that their expression patterns are tissue-specific, whereas PERK, ATF6, and IRE1 expression is ubiquitous. Target sequences of their active N-terminal fragments are different among family members.

BBF2H7 is an ER stress transducer essential for differentiation and proliferation of chondrocytes. Active N-terminal fragments of BBF2H7 bind to cAMP response element (CRE)-like sequences in the promoter regions of the *Sec23a* gene and enhance its expression [87]. SEC23 is a component of COPII vesicles essential for ER-Golgi transport. Correspondingly, in Bbf2h7 KO mice, the number of proliferating chondrocytes is significantly reduced [87]. Recently, it was found that Bbf2h7 is directly regulated by Sox9, which is a master regulator of chondrocyte differentiation [88]. In humans, a homozygous F382L missense mutation in the *SEC23A* gene was identified in the family affected with cranio-lenticulo-sutural dysplasia, which is a rare autosomal recessive syndrome characterized by late-closing fontanels, sutural cataracts, facial dysmorphisms, and skeletal defects [89]. Fibroblasts of affected patients showed greatly distended ER cisternae, suggesting that ER-Golgi COPII vesicle transportation was disturbed [89,90,91].

OASIS is also essential for differentiation and proliferation of osteoblast. Similar to BBF2H7, active N-terminal fragments of OASIS binds to CRE-like sequences in the promoter regions of the *Col1a1* gene, and enhance its expression [92,93]. *Col1a1* encodes the main component of type I collagen, which is a major bone matrix, and therefore, OASIS KO mice display severely impaired osteogenesis [92]. In humans, homozygous genomic deletion of CREB3L1, which encodes OASIS, was detected in Turkish patients with severe osteogenesis imperfecta, which is a heterogenous congenital disorder characterized by increased bone fragility [94]. The more prevalent autosomal dominant forms of osteogenesis imperfecta are caused by mutations in the *COL1A1* and *COL1A2* genes, which encode the α1- and α2-chains of type I procollagen, respectively [95].

BBF2H7 and OASIS are susceptible to the ubiquitin-proteasome system-mediated protein degradation in the absence of ER stress, but are stabilized by dissociation from ubiquitin E3 ligase HRD1 in response to mild ER stress, which is triggered by differentiation of chondrocytes and osteoblasts [96]. In the intestine, it was shown that OASIS was required for differentiation of goblet cells, and mucin secretion machinery [97].

BBF2H7 and OASIS are unique in that they respond to mild and physiological ER stress, and have pivotal roles in tissue differentiation and proliferation [98]. It has been conceptually accepted that ER stress is toxic to cells and that ER stress-mediated cell death is associated with human diseases; however, the discovery of the OASIS family has led to the recognition that “physiological” ER stress is necessary for normal tissue generation and differentiation [99].

## 3. ER Stress and Endocrine Disorders

In this section we discuss recent findings on ER stress and human endocrine disorders.

### 3.1. Diabetes Mellitus

Diabetes mellitus is a chronic disease associated with abnormally high blood glucose levels, and occurs when pancreatic β-cells fail to meet insulin demand due to loss of β-cell mass and function. As described previously, pancreatic β-cells have to adapt to insulin-dependent increases in secretory demand. It is unlikely that UPR is involved in the release of insulin because mature insulin molecules are stored within the secretory granules and secreted depending on serum glucose levels. The cause of ER stress in pancreatic β-cells is proinsulin production, which is shown to physiologically stimulate pancreatic β-cell proliferation [100]. However, large ER stress, which overwhelms UPR because of several pathological situations such as insulin resistance or mutant proinsulin molecule, finally causes loss of β-cell mass and function. Perk KO mice display early-onset diabetes because of impaired insulin secretion by pancreatic β-cell apoptosis [101], and Chop KO mice ameliorate the diabetic phenotype of Akita mice, which are susceptible to ER stress by a mutant insulin molecule [102] (see below). Furthermore, in humans, mutations in the *EIF2AK3* gene, which encodes PERK, causes Wolcott–Rallison syndrome, which is characterized by permanent neonatal or early infancy diabetes because of impaired insulin secretion [103]. These studies led many researchers to focus on the link between ER stress and diabetes. In pancreatic β-cells of Atf6α KO mice, UPR was shown to be activated, which leads to impaired proinsulin production under a high-fat diet [104], and pancreatic β-cell specific Ire1α conditional KO mice have diabetes under a normal diet [105]. Similarly, pancreatic β-cell specific Xbp1 conditional KO mice display impairment of proinsulin production, proinsulin processing, and insulin secretion because of defective ER membrane proliferation [106]. These findings indicate that all three major pathways are involved in ER stress of pancreatic β-cells.

Perk KO mice display early-onset diabetes, which suggests the PERK pathway has a cytoprotective role by attenuating proinsulin biosynthesis, but in contrast, it has been demonstrated that GADD34 and CReP constitutively suppress phosphorylation of eIF2α and enhance proinsulin production [107]. In contrast to the cytoprotective characteristics of IRE1α described above, sustained exposure to high blood glucose was shown to trigger RIDD-mediated proinsulin mRNA decay in pancreatic β-cells, which leads to a vicious cycle of glucotoxicity [77,108].

There are several mouse models of diabetes. A famous ER stress-mediated diabetic model, Akita mice, has a C96Y heterozygous missense mutation in the *Ins2* gene, which encodes insulin [109]. C96Y mutant insulin replaces a cysteine residue that is engaged in the formation of an intramolecular disulfide bond between the A and B chains of the proinsulin molecule, which leads to an aberrant structure of mutant proinsulin and ER stress [102,110]. In insulinoma cell lines established from pancreatic β-cells of Akita mice, the ATF6 and IRE1XBP1 pathways are constitutively activated [111]. In Akita mice, the transcription factor C/EBPβ accumulates in the islets as a result of ER stress before onset of hyperglycemia, which results in reduced BiP synthesis through decreased ATF6 activity, and consequently, vulnerability to ER stress and apoptosis [112]. In humans, several *INS* gene mutations, including the C96Y missense mutation, are causes of neonatal diabetes [113], whereas Akita mice may be considered to be a model of MODY in humans.

Mice with a heterozygous S51A mutation in *Eif2a*, which encodes eIF2α, at the eIF2α phosphorylation site display diabetes under a high-fat diet, whereas mice homozygous for the S51A mutation are embryonic lethal or die within 18 h after birth because of hypoglycemia associated with defective gluconeogenesis by severe wasting syndrome [114]. 58-kDa inhibitor of protein kinase (P58IPK) is required for PERK inhibition, and P58IPK KO mice display more severe diabetes crossing with an Akita *Ins2* mutant [115]. Thus, PERK signaling has a pivotal role in glucose homeostasis. However, it should be noted that these mice models carrying specific mutations in genes involved in UPR may not precisely demonstrate human diabetic conditions because the gene mutations are artificial and very rare in humans.

### 3.2. Obesity (Adiponectin and Leptin)

Obesity is defined as abnormal or excessive fat accumulation that may impair health. Obesity is linked to metabolic disorders such as insulin resistance and atherosclerosis. Obesity results in chronic ER stress in adipose tissue, which is triggered by the release of nonesterified fatty acid (NEFA) [116]. ER stress in adipose tissue induces inflammation via the PERK-eIF2α pathway, which leads to increased proinflammatory cytokine expression such as TNF-α and IL-6 in a loop manner [117].

Adiponectin is an insulin sensitizer with anti-insulin resistance and anti-inflammatory properties [118]. Impaired adiponectin expression contributes to development of insulin resistance [119]. Obesity-mediated hypoxic environments in adipocytes causes ER stress, which transcriptionally downregulates adiponectin by negative regulatory effect of CHOP which binds to adiponectin promoter [120]. Also, adiponectin expression is negatively regulated by ER stress-mediated ATF3 [121]. ER stress-mediated autophagy has been shown to degrade adiponectin [122]. Adiponectin multimerization is important for its secretion and function, and disulfide bond A oxidoreductase-like protein (DsbA-L) has a pivotal role in the multimerization and stability of adiponectin [123,124,125]. ER stress-inducible ATF3 described above also contributes to adiponectin resistance by negatively regulating adiponectin receptor 1 expression [126], and adiponectin receptor 2 expression [127]. Adiponectin is shown to have a protective effect from ER stress-mediated cell death [128,129], and thus adiponectin resistance may cause high levels of ER stress in chronic metabolic diseases.

Leptin is an adipocyte-derived hormone that suppresses appetite mainly through its action on a subset of hypothalamic neurons [130]. It has been shown that leptin expression is also downregulated by ER stress. ER stress-induced CHOP negatively regulates C/EBPα, which is required for leptin transcription [131]. In the majority of the obese population, leptin resistance is prevalent and contributes to increased appetite and reduced energy expenditure. Obese-induced ER stress is associated with inhibition of leptin receptor signaling, and improved ER capacity enhances leptin sensitivity, suggesting that ER stress plays an important role in the development of leptin resistance [132]. This leptin resistance is caused by downregulation of mitofusin 2, which plays fundamental roles in both mitochondrial fusion and mitochondria–ER interaction [133].

The ob mutation is located in the gene encoding leptin, and mice homozygous for the ob mutation (*ob/ob* mice) become hyperphagic, insulin resistant, and hyperinsulinemic [134]. In *ob/ob* mice, the ER stress response in the liver is activated and Xbp1 KO mice develop insulin resistance, suggesting that ER stress is involved in obesity and insulin resistance [135]. Correspondingly, expression of ER stress response genes is upregulated in the adipose tissue of obese patients [136]. OPR150 is a molecular chaperone located in the ER and its expression is upregulated by both ER stress and oxidative stress. Systemic expression of ORP150 ameliorates diabetes of Akita mice through reduced insulin resistance [137,138].

### 3.3. Central Diabetes Insipidus (CDI)

CDI is a disorder in which the patient excretes a large volume of hypotonic and dilute urine due to impaired secretion of AVP. AVP is produced in the hypothalamus, and is stored in the posterior pituitary gland. AVP is the main hormone involved in the regulation of water homeostasis and osmolality. AVP binds to V_2_ receptors found on renal collecting duct epithelia, and enhances water reabsorption. AVP is synthesized as part of a precursor molecule, AVP-NPII, packaged in neurosecretory granules, and cleaved to AVP during transport to the posterior pituitary. The AVP-NPII protein has a complex structure including seven disulfide bonds, and therefore, gene mutations that encode NPII mainly alter AVP-NPII protein structure and cause ER stress by ER-localized mutant AVP-NPII protein. As a result, impaired AVP secretion causes familial neurohypophyseal diabetes insipidus (FNDI).

Several studies have demonstrated ER stress involvement in FNDI. Although not different between ATF6α KO mice and wild-type mice, urine volumes in ATF6α KO mice are increased by intermittent water deprivation (WD) through impaired AVP secretion, a finding that indicates the ATF6 pathway has an important role in AVP synthesis and secretion [139]. In addition, another group created FNDI model mice that have a C98X heterozygous nonsense mutation at the endogenous *Avp* locus. Electron micrographs revealed protein aggregates in the ER of the hypothalamus in these mice [140]. Furthermore, under WD conditions, protein aggregates in the ER are scattered and are involved in autophagy, which leads to autophagic cell death [141]. These findings indicate that both ER stress and autophagy are involved in impaired AVP synthesis and secretion in FNDI. It has been shown that the UPR can regulate autophagy in protein misfolding disorders [142]. ATF4 activates several autophagy-related genes involved in the formation, elongation and function of the autophagosome in the adaptation to stress [19,143], whereas it is still elusive that similar mechanisms are involved in FNDI. Interestingly, in these model mice, shortening of the RNA polyA tail was reported, which increased mRNA turnover and resulted in decreased amounts of Avpnp mRNA. It was demonstrated that this decrease in mRNA was not nonsense mRNA decay because of the C98X nonsense mutation, and, furthermore, that wild-type Avpnp mRNA was also implicated in this novel mechanism [10,144].

Gene mutations that encode AVP can potentially cause ER retention of mutant AVP-NPII [145]. The novel ER stress transducer described previously OASIS was shown to bind to the promoter region of the AVP-NPII gene and regulate gene expression in the hypothalamus [146].

### 3.4. Wolfram Syndrome

Wolfram syndrome is a rare genetic disorder characterized by juvenile-onset diabetes mellitus with impaired insulin secretion, hearing loss, central diabetes insipidus, optic nerve atrophy, and neurodegeneration. The majority of patients have recessive mutations in the *WFS1* gene [147]. WFS1 is localized on the ER membrane, and ER stress enhances its expression, which suggests mutations in *WFS1* may render cells vulnerable to ER stress that leads to cell death and onset of Wolfram syndrome [148,149]. WFS1 directly binds to ATF6 and enhances ATF6 degradation by the ubiquitin-proteasome system with the assistance of the ubiquitin E3 ligase, HRD1. Presumably, this mechanism protects cells from overactivation of the ATF6 pathway, which leads to apoptosis [150].

Although this disorder usually demonstrates autosomal recessive inheritance, it should be noted that some heterozygous *WFS1* mutation carriers have an increased risk of low frequency sensorineural hearing loss (LFSNHL) [151] in addition to an increased risk of depression and suicide [152]. Furthermore, patients heterozygous for the H313Y mutation in *WFS1* exceptionally exhibit Wolfram syndrome in an autosomal dominant manner [153,154].

Pancreatic β-cell-specific Wfs1 conditional KO mice do not display overt diabetes, whereas IPGTT showed significant glucose intolerance compared to littermate controls [155]. However, transplanting insulin-producing cells established from iPS cells of patients with Wolfram syndrome to immunodeficient mice, glucose intraperitoneal administrations revealed significantly lower insulin responses in Wolfram β-cells [156].

### 3.5. Isolated Growth Hormone Deficiency Type II (IGHD2)

IGHD2 is congenital GH deficiency inherited as an autosomal dominant trait [157]. GH is secreted by somatotrophs, and is essential for longitudinal bone growth. Abnormalities in *GH1*, which encodes GH, cause short stature because of impaired GH secretion. One *GH1* allele appears sufficient to maintain normal GH secretion because patients harboring a deletion in one *GH1* allele exhibit normal stature [158]. However, patients with heterozygous intron 3 splice-site mutations in *GH1* demonstrate impaired GH secretion despite having a wild-type *GH1* [159,160]. This disorder is termed isolated growth hormone deficiency type II (IGHD2), and it has been suggested that the mutant allele exerts a dominant negative effect on the secretion of wild-type GH, but the precise molecular mechanisms involved have yet to be elucidated.

In IGHD2, splice-site mutations in intron 3 cause in-frame mRNA skipping of exon 3, which corresponds to amino acids 32 to 71, and leads to production of a 17.5 kDa exon 3 deletion-mutant GH protein (del32-71 GH). It is widely accepted that the most likely explanation for the mechanism of the dominant negative effect are protein interactions between wild-type and del32-71 GH, such as heterodimers [161,162,163,164,165]. However, no studies to date have shown wild-type and del32-71 GH heterodimers. On the other hand, del32-71 GH localizes to the ER because of its aberrant protein structure and is degraded by ERAD [164], which suggests del32-71 GH may cause ER stress. While involvement of ER stress in the dominant negative effect of del32–71 GH has been speculated to be unlikely [166,167], we have previously demonstrated that del32-71 GH caused ER stress-mediated cell death in vitro [168].

In del32-71 GH transgenic mice, the sole existing murine model for IGHD2, extensive pituitary damage by macrophage invasion was demonstrated [167]. It is still unclear whether protein interaction by both wild-type and del32-71 GH or ER stress by del32-71 GH was responsible for the pituitary damage. Using this murine model, several in vivo studies were performed, including del32-71 GH knockdown by shRNA [169] and altering splicing efficiency using butyrate [170], which successfully ameliorated GH impaired secretion. However, the underlying mechanisms have not been uncovered in vivo. In del32-71 GH transgenic mice, the entire *GH1* locus control region was used for copy number-dependent del32-71 GH expression. Therefore, the mouse line demonstrating severe pituitary damage may have had supraphysiological expression of del32-71 GH. The growth curve of del32-71 GH transgenic mouse lines showed severe growth retardation comparable to that found in Ghr KO mice, which had null GH activity [167,171]. In contrast, some patients with IGHD2 exhibit a mild GH deficiency, which has been clinically undetected until they reached their final height. Theoretically, murine models in which human wild-type and mutant *GH1* are knocked-in with a single copy each (replacing the endogenous mouse *Gh* gene) may precisely demonstrate the observed phenotypes found in patients with IGHD2 and be a powerful tool to uncover the dominant negative effect of del32-71 GH in vivo.

## 4. Treatment of ER Stress-Related Diseases

In this review article, we described the role of ER stress in endocrine disorders. On a final note, we discuss possible treatment of ER stress-related diseases. As described previously, unfolded proteins accumulated in the ER give rise to ER stress when they overwhelm available folding capacity. Thus, compounds that increase folding capacity for unfolded proteins and work as chemical chaperones are good therapeutic candidates.

Several studies have investigated increases of folding capacity. Pre-conditioning of the PERK pathway was reported to be cytoprotective to ER stress [172], but it is unclear whether similar effects are also found in vivo. BiP inducer X (BIX) was identified that selectively induces BiP expression downstream of ATF6 [173]. Using osteoporotic ovariectomized mice, it was found that oral administration of BIX effectively slowed the decline in bone formation through folding activation and secretion of bone matrix proteins [174]. In addition, BIX was reported to effectively prevent neuronal cell death by brain ischemia [175,176]. Artificially sustained IRE1α activity was reported to enhance cell survival [177,178]. Using the ATP-competitive inhibitor 1NM-PP1, it was demonstrated that RNase activity was artificially induced, which leads to cytoprotective XBP1 transcription [179].

In addition, chemical chaperones were reported to enhance protein folding [180]. Currently, sodium phenylbutyrate (4PBA) is used as an ammonia scavenger for pediatric and adult patients with urea cycle disorders [181], and several studies have demonstrated that 4PBA ameliorates ER stress-mediated cell toxicity [182,183]. Tauroursodeoxycholic acid (TUDCA) is another well-characterized chemical chaperone that mitigates ER stress [184,185]. Also, recently, azoramide was shown to regulate ER folding and secretion capacity without inducing ER stress [186].

Several studies have shown other currently used therapeutic agents with demonstrated effectiveness in ER stress-related diseases. Naratriptan, which is used for migraines, decreases CGRP1 expression induced by mutant androgen receptor, and ameliorates the neurological symptoms in spinal and bulbar muscular atrophy model mice [187]. Valproate, which is a widely used antiepileptic drug, mitigates ER stress caused by the trans-activation responsive region (TAR) DNA-binding protein in a model of amyotrophic lateral sclerosis in mice [188].

## 5. Conclusions

Endocrine cells are susceptible to ER stress because of the necessity to adapt to rapid increases in protein demand, and because of the complicated protein structure of hormones, which includes disulfide bonds. In addition to conventional ER stress sensors, novel ER stress transducers, the OASIS family, have been recently described. The OASIS family members are unique in that they are activated in the presence of a mild physiological ER stress. Therefore, in at least some tissues, ER stress is mandatory for normal tissue differentiation and proliferation.

Diabetes mellitus, central diabetes insipidus, and Wolfram syndrome are caused by ER stress-mediated apoptosis. In addition to these disorders, IGHD2 was recently found to be associated with ER stress. The molecular mechanism that underlies the dominant negative effect caused by del32-71 GH is not known, and establishment of an animal model that precisely demonstrates the phenotypes found in patients with IGHD2 is necessary.

Recently, chemical chaperones have attracted attention for treatment of ER stress-related diseases. In particular, 4PBA can be safely administered to pediatric and adult patients, and it was reported to ameliorate ER stress in an animal model.
